# The 9p21 susceptibility locus for coronary artery disease and the severity of coronary atherosclerosis

**DOI:** 10.1186/1471-2261-9-3

**Published:** 2009-01-27

**Authors:** Suet Nee Chen, Christie M Ballantyne, Antonio M Gotto, Ali J Marian

**Affiliations:** 1 Center for Cardiovascular Genetics, Brown Foundation Institute of Molecular Medicine, The University of Texas Health Science Center, and Texas Heart Institute, Houston, TX 77030, USA; 2 Graduate Program in Cardiovascular Sciences, Baylor College of Medicine, Houston, TX, USA; 3 Section of Atherosclerosis and Vascular Medicine, Department of Medicine, Baylor College of Medicine, and Methodist DeBakey Heart and Vascular Center, Houston TX, USA; 4 Weil College of Medicine of Cornel University, New York, NY, USA

## Abstract

**Background:**

Case-control Genome-Wide Association Studies (GWAS) have identified single nucleotide polymorphisms (SNPs) at the 9p21 locus as risk factors for coronary artery disease (CAD). The locus does not contain a clear candidate gene. Hence, the results of GWAS have raised an intense interest in delineating the basis for the observed association. We analyzed association of 4 SNPs at the 9p21 locus with the severity and progression of coronary atherosclerosis, as determined by serial quantitative coronary angiograms (QCA) in the well-characterized Lipoprotein Coronary Atherosclerosis Study (LCAS) population. The LCAS is a randomized placebo-control longitudinal follow-up study in patients with CAD conducted to test the effects of fluvastatin on progression or regression of coronary atherosclerosis.

**Methods:**

Extensive plasma lipid levels were measured at the baseline and 2 1/2 years after randomization. Likewise serial QCA was performed at the baseline and upon completion of the study. We genotyped the population for 4 SNPs, previously identified as the susceptibility SNPs for CAD in GWAS, using fluorogenic 5' nuclease assays. We reconstructed the haplotypes using Phase 2, analyzed SNP and haplotype effects using the Thesias software as well as by the conventional statistical methods.

**Results:**

Only Caucasians were included since they comprised 90% of the study population (332/371 with available DNA sample). The 4 SNPs at the 9p21 locus were in tight linkage disequilibrium, leading to 3 common haplotypes in the LCAS population. We found no significant association between quantitative indices of severity of coronary atherosclerosis, such as minimal lumen diameter and number of coronary lesions or occlusions and the 9p21 SNPs and haplotypes. Likewise, there was no association between quantitative indices of progression of coronary atherosclerosis and the SNPs or haplotypes. Similarly, we found no significant SNP or haplotype effect on severity and progression of coronary atherosclerosis.

**Conclusion:**

We conclude the 4 SNPs at the 9p21 locus analyzed in this study do not impart major effects on the severity or progression of coronary atherosclerosis. The effect size may be very modest or the observed association of the CAD with SNPs at the 9p21 locus in the case-control GWAS reflect involvement of vascular mechanisms not directly related to the severity or progression of coronary atherosclerosis.

## Background

Genome-wide association studies (GWAS), wherein the alleles and genotypes frequencies of a large number of single nucleotide polymorphisms (SNPs) in cases with the phenotype, and in controls are compared, have emerged as the state of the art approach to identify the susceptibility alleles for the common complex traits. Recently, several large-scale GWAS have shown a very strong association between coronary artery disease (CAD) and SNPs at the 9p21 locus [1-4]. The observed association of CAD or surrogate phenotypes with SNPs at the 9p21 locus has been replicated and confirmed in a very large number of populations, an important step toward validation of the results of any association study [5-12]. The findings have raised considerable interest in identifying the putative candidate susceptibility gene at the 9p21 locus, particularly considering that the region is sparse in genes. It is comprised of only two known genes, namely, *CDKN2A *[a.k.a. Alternative Reading Frame variant (*ARF*)] and *CDKN2B*, which encode tumor suppressor proteins p16INK2a and p16INK2b, respectively. The 9p21 locus is frequently lost or mutated in various human cancers [13-15]. Likewise, germ-line deletion of the locus in the mouse leads to tumorigenesis but is not known to predispose to or protect from atherosclerosis [16,17]. The locus also contains a non-coding RNA gene with yet to be defined function. Therefore, the weight of the very strong genetic association is somewhat offset by the lack of a clear biologically plausible candidate gene at the mapped locus. Thus, there is a heightened interest in delineating the basis for the reproducible results of case-control GWAS and the putative mechanisms by which the 9p21 locus predisposes to CAD.

Detailed phenotyping is a crucial step not only in design and interpretation of the results of any phenotype-genotype association study, but also in guiding the subsequent studies to delineate the molecular mechanisms pertaining to the observed association. Since the severity of atherosclerosis is a continuum, it would be expected that the quantitative indices of severity of coronary atherosclerosis to provide more robust analytic results than a dichotomous definition used in the case-control GWAS studies. Accordingly, a previous study analyzed association of the SNPs at the 9p21 locus with coronary calcium score on computerized tomography as a quantitative surrogate index for coronary atherosclerosis [5]. Coronary calcium score, however, was analyzed as a categorical phenotype and allele frequencies were compared in those with 0, 1–100 and > 100 coronary calcium scores [5]. In the present study, we used quantitative coronary angiography (QCA), one of the most robust and commonly used method for accurate quantification of severity of coronary atherosclerosis, to assess the severity of coronary atherosclerosis [18]. Therefore, we hypothesized that the SNPs at the 9p21 locus, implicated as susceptibility SNPs in the case-control GWAS [1-4], are associated with the baseline severity as well as progression of coronary atherosclerosis, as determined by QCA. To test this hypothesize, we determined association of quantitative indices of severity of coronary atherosclerosis as well as progression of coronary atherosclerosis over a 2 1/2 year period with 4 SNPs at 9p21 locus in a well characterized Caucasian population.

## Methods

### Study Population

332 Caucasian individuals who participate in the Lipoprotein and Coronary Atherosclerosis Study (LCAS) comprised the study population. The study design and the main results of the LCAS have been published [19]. In brief, 429 males and females between the ages of 35 to 75 years who had at least 1 coronary lesion causing 30% to 75% luminal diameter stenosis on QCA and had a plasma low-density lipoprotein-cholesterol (LDL-C) level of 115 to 190 mg/dl despite diet participated in LCAS. DNA samples were available in 371 subjects. To avoid the potential confounding effects of ethnic background and given that the GWAS of CAD were performed mostly in Caucasians, we included only Caucasians, who comprised 90% of the study population (N = 332). We measured total cholesterol, LDL-C, high-density lipoprotein-cholesterol (HDL-C), triglyceride (TG), lipoprotein (a) (Lp [a]), and apolipoprotein levels in all participants and quantitative indices of severity of coronary atherosclerosis in 288 participants at the baseline. We then randomized the participants to treatment with either fluvastatin (40 mg daily) or placebo and repeated the detailed phenotyping after 2.5 years of therapy. The study protocol was approved by the institutional review board, and all participates were provided with written informed consent that included genetic studies.

### Genotyping

In the case-control GWAS, 4 SNPs, namely rs2383206 (chromosomal position: 22,105,026), rs2383207 (chromosomal position: 22,105,959), rs10757278 (chromosomal position: 22,114,477), rs1333049 (chromosomal position: 22,115,503) were the primary risk SNPs at the 9p21 locus [1-4]. All chromosomal positions were per reference assembly (build 36.3). We genotyped the putative risk SNPs by fluorogenic 5' nucleotidase (Taqman) assays using an ABI PRISM^® ^7900 HT Real-Time PCR instrument. The PCR conditions were 1 cycle at 95°C for 10 min, followed by 40 cycles of 92°C for 15 sec and 60°C for 1 min as recommended by the manufacturer (Applied Biosystems, Foster City, CA). An investigator who had no knowledge of the angiographic and clinical data performed the genotyping.

### Linkage disequilibrium (LD), haplotype reconstruction and statistical genetics

We estimated LD between the SNPs by the expectation maximization (EM) algorithm using Arlequin 2.0 [20]. Deviation from Hardy-Weinberg equilibrium was estimated by χ^2 ^test. We used PHASE 2.1 to reconstruct and determine frequencies of the haplotypes. It implements Bayesian statistical method to infer phase and reconstruct haplotypes from population genetics by the Markov Chain-Monte Carlo algorithm and coalescent theory, with partition ligation algorithm. We detected the haplotype effect size using the Thesias program, which is based on the maximum likelihood model and is linked to the stochastic version of the expectation maximization algorithm [21,22].

### Conventional statistical analysis

We expressed the continuous variables as mean ± SD and determined variance equality by the Bartlett's test. We compared the differences among the non-parametric variables with equal variances among the three genotypes by the analysis of variance followed by pair wise comparisons (Bonferroni method). We compared the differences among the phenotypes with unequal variances by the Kruskal-Wallis test. We analyzed the differences in the parametric variables among the three genotypes by Chi Square test. We also performed multivariate robust and logistic regression analysis to detect independent association of the SNPs and demographic variables with indices of severity of coronary artery disease and its progression. We used a general genetic model by assigning indicators 0, 1 and 2 to the genotypes and avoided assuming a dominant or a recessive effect in the absence of biological evidence. Statistical analysis was performed using STATA program v9.2.

## Results

### Baseline characteristics of the study population

Demographics, plasma lipid levels and indices of severity of coronary atherosclerosis at the baseline are shown in Table 1.

**Table 1:** Demographics, Baseline Plasma Lipid Levels and Indices of Severity of Coronary Atherosclerosis

**A. Demographics (**N = 332)	
Age (years)	59.3 ± 7.7

Male/Female	280 (84%)/52 (16%)

Height (m)	1.74 ± 0.08

Body Weight (kg)	84.85 ± 15.62

Body mass index (kg/m^2^)	28.06 ± 4.50

Waist/Hip	0.91 ± 0.68

Systolic blood pressure (mmHg)	124.48 ± 15.31

Diastolic blood pressure (mmHg)	76.67 ± 8.82

Previous myocardial infarction (%)	130 (39%)

Diabetes mellitus (%)	11 (3.3%)

Current smokers (%)	68 (20%)

**B. Plasma Lipid levels **(N = 332)	

Total Cholesterol (mg/dl)	220.0 ± 24.5

HDL-C (mg/dl)	43.9 ± 11.3

LDL-C (mg/dl)	143.9 ± 19.9

LDL-C/HDL-C	3.49 ± 0.91

Triglyceride (mg/dl)	161.5 ± 57.1

Apo A1 (mg/dl)	132.7 ± 27.4

Apo B (mg/dl)	134.4 ± 20.8

ApoB/ApoA1	1.05 ± 0.26

Apo C-III (mg/dl)	37.5 ± 12.3

Lipoprotein (a) (mg/dl)	132.7 ± 27.4

**C. Quantitative Indices of Severity of Atherosclerosis **(N = 288)	

Number of coronary lesions (mean)	2.99 ± 1.37

Number of subject with ≥ one coronary lesion (%)	287 (86%)

Number of coronary occlusions (mean)	0.30 ± 0.55

Number of subject with ≥ one coronary occlusion (%)	83 (25%)

Average baseline MLD (mm)	1.68 ± 0.40

**Abbreviations: **m: meter; Kg: Kilogram; mmHg: millimeter mercury; mg/dl: milligram per deciliter; HDL-C: High-density lipoprotein-cholesterol; LDL-C: low-density lipoprotein-cholesterol; Apo A: apolipoprotein A; Apo B: apolipoprotein B; Apo C-III: apolipoprotein C3; MLD: minimal lumen diameter.

### Linkage disequilibrium and haplotypes

**Figure 1:**
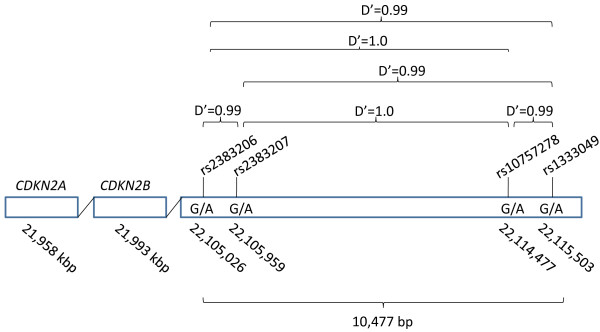
**Chromosomal positions of the 4 SNPs analyzed and linkage disequilibrium (LD)**. The chromosomal positions of rs2383206, rs2383207, rs10757278, rs1333049 according to reference assembly are shown along with the D' values indicating near perfect LD among the 4 SNPs. The positions of the 3 adjacent genes are also shown, none of which is a biologically plausible candidate gene for coronary atherosclerosis or its thrombotic complications.

We selected the rs2383206, rs2383207, rs10757278 and rs1333049 because they were associated with the risk of ischemic heart disease/CAD in the case-control GWAS [1-4]. The GG genotype and the G allele frequencies were higher in the cases than in the controls in GWAS, the latter by approximately 5% [1-4]. The frequency of the G allele (rs238206) in the LCAS population was 0.551, which is largely in accord with the observed frequency in the US population [3]. The position of the SNPs on the references assembly and LD between the SNPs in the LCAS population are shown in Figure 1. The 4 SNPs spanned 10,477 bp of DNA at the 9p21 locus and encompassed 11 additional SNPs listed in the HapMap database. All SNPs were in strong LD with each other and also captured all of the 11 other alleles in this region with a mean maximum r^2 ^of 0.932. As would be expected, there were no significant differences in genotype-phenotype association among the 4 SNPs. Therefore, data for the phenotype-genotype association is shown only for rs238206 SNP (phenotypic data for 3 other SNPs are available upon request). Haplotype reconstruction using Phase 2.1 showed 3 common haplotypes of G-G-G-A, *f *= 0.506; A-A-A-G, *f *= 0.442 and G-G-A-G, *f *= 0.038) and 5 rare haplotypes (detail data available upon request).

### Genotype-phenotype association

Demographic characteristics, plasma levels of lipid and angiographic indices of severity of coronary atherosclerosis at the baseline were not significantly different among the genotypes of any of the 4 SNPs analyzed. The results for the rs238206 SNP are shown in Table 2. Likewise, as would be expected because of strong LD among the 4 SNPs, we detected no significant haplotype effect on quantitative indices of severity of coronary atherosclerosis (data available upon request).

**Table 2:** Clinical Phenotypes and Genotypes of rs2383206 SNP at the 9p21 Locus

	**Genotypes**			
	**GG**	**GA**	**AA**	**P**

**N-332**	**88**	**190**	**54**	

**A. Demographics**				

Age	58.57 ± 7.89	59.42 ± 7.48	60.11 ± 7.75	0.482

Male/Female	72/16	161/29	47/7	0.689

Height (m)	1.73 ± 0.09	1.74 ± 0.08	1.73 ± 0.08	0.462

Body weight (kg)	84.12 ± 15.88	85.43 ± 16.11	84.00 ± 13.68	0.737

BMI (Kg/m^2^)	27.97 ± 4.34	28.08 ± 4.59	28.12 ± 4.50	0.976

Hip/Waist	1.11 ± 0.09	1.10 ± 0.09	1.11 ± 0.09	0.649

Systolic BP (mmHg)	125.34 ± 15.18	124.03 ± 15.65	124.70 ± 14.55	0.797

Diastolic BP (mmHg)	77.64 ± 8.59	76.34 ± 8.64	76.30 ± 9.37	0.492

Previous MI (%)	35(39.75)	73(38.4)	22(40.7)	0.945

Diabetes Mellitus (%)	5 (1.5)	5 (1.5)	1 (0.3)	0.337

Current smoker (%)	19 (21.6)	40 (21.1)	9 (16.7)	0.746

**B. Plasma Lipid levels**				

Total Cholesterol (mg/dl)	222.05 ± 23.74	220.14 ± 23.98	216.19 ± 23.74	0.384

HDL-C (mg/dl)	44.27 ± 12.85	43.69 ± 11.24	43.80 ± 8.72	0.716*

LDL-C (mg/dl)	144.78 ± 19.74	144.02 ± 20.08	144.78 ± 19.74	0.750

LDL-C/HDL-C	3.53 ± 0.97	3.50 ± 0.92	3.39 ± 0.79	0.647

Triglyceride (mg/dl)	165.20 ± 64.87	162.33 ± 55.60	151.23 ± 48.01	0.691*

Apo A1 (mg/dl)	136.03 ± 31.37	131.81 ± 27.20	130.56 ± 20.40	0.619*

Apo B (mg/dl)	137.19 ± 22.16	134.17 ± 20.36	130.31 ± 19.44	0.157

ApoB/ApoA1	1.05 ± 0.26	1.06 ± 0.26	1.02 ± 0.23	0.666

Apo C-III (mg/dl)	37.95 ± 13.36	38.28 ± 12.25	33.67 ± 9.85	0.0465

Lipoprotein (a) (mg/dl)	40.07 ± 37.38	33.34 ± 31.98	33.87 ± 27.08	0.253*

**C. Quantitative Indices of Severity of Atherosclerosis**				

**N **= 288	77	162	49	

Number of coronary lesions (mean)	3.09 ± 1.43	2.93 ± 1.37	3.06 ± 1.30	0.639

≥ 1 coronary lesion (%)	77 (87.5)	161 (84.7)	49 (90.7)	0.495

Number of coronary occlusions(mean)	0.34 ± 0.59	0.27 ± 0.54	0.30 ± 0.50	0.636

≥ 1 coronary occlusion (%)	25 (28.4)	43 (22.6)	15 (27.8)	0.513

Baseline MLD (mm)	1.65 ± 0.39	1.69 ± 0.40	1.68 ± 0.40	0.819

**Abbreviations: **BMI: Body mass índex; BP: Blood pressure; MI: Myocardial infarction; other abbreviations are as in Table 1

* indicates p value by non-parametric Kruskal-Wallis test

### Genotypes and progression of coronary atherosclerosis

There were no significant differences in the mean number of new coronary lesions or coronary occlusions and the number of individuals who developed new lesions or new occlusions during the 2.5-year follow up period among the genotypes (Table 3). Similarly, there was no significant haplotype effect on progression of coronary atherosclerosis (data available upon request).

**Table 3:** Progression (Regression) of Atherosclerosis Over a 2.5-Year Period (in the Placebo Group) According to the Genotypes of rs2383206 SNP at the 9p21 Locus

	**Genotypes**			
	**GG**	**GA**	**AA**	**P**

**N **= 141	32	78	31	

New coronary lesion (mean)	0.34 ± 0.48	0.38 ± 0.49	0.26 ± 0.45	0.462

Subjects with new coronary lesions (%)	11 (34.3)	30 (38.4)	8 (25.8)	0.265

New coronary occlusions (mean)	0.03 ± 0.18	0.08 ± 0.27	0.06 ± 0.25	0.678

Subjects with new coronary occlusions (%)	1 (3.1)	6 (7.7)	2 (6.5)	0.209

Average final MLD (mm)	1.54 ± 0.40	1.62 ± 0.41	1.59 ± 0.39	0.657

Change in MLD (mm)	-0.09 ± 0.25	-0.11 ± 0.23	-0.11 ± 0.25	0.928

**Abbreviations: **m: meter; Kg: Kilogram; mmHg: millimeter mercury; mg/dl: milligram per deciliter; HDL-C: High-density lipoprotein-cholesterol; LDL-C: low-density lipoprotein-cholesterol; Apo A: apolipoprotein A; Apo B: apolipoprotein B; Apo C-III: apolipoprotein C3; MLD: minimal lumen diameter.

### Genotype and response to fluvastatin therapy

Treatment with fluvastatin reduced plasma LDL-C levels by 25.7% (p < 0.0001), raised HDL-C by 9.3% (p < 0.0003) and slowed progression of atherosclerosis (MLD loss -0.04 mm vs. -0.11 mm in the placebo, p = 0.009). However, there were no genotype-by-treatment interactions in changes in plasma levels of lipid among the genotypes, with the exception of plasma levels of triglycerides, which showed a significant genotype-dependent change (for all 4 SNPs). Likewise, changes in quantitative indices of severity of coronary atherosclerosis were not significantly different among the genotypes of the 4 SNPs. Data for changes in the plasma lipid levels and indices of severity of atherosclerosis according to the genotypes of rs238206 SNP are shown in Table 4. Finally, only treatment with fluvastatin (regression coefficient: 0.082, 95% CI: [0.028–0.136], t = 3.02, p = 0.003) and plasma HDL-C levels at the baseline (regression coefficient: 0.004, 95% CI: [0.002 – .006], t = 3.51, p = 0.001) were the only independent predictors of progression of coronary atherosclerosis (ΔMLD during the follow up) in robust regression analysis but not any of the 4 SNPs or the demographic variables sex, age, body mass index, diabetes, smoking, alcohol consumption and the baseline plasma levels of LDL-C and triglyceride.

**Table 4:** Response of Plasma Lipid and Coronary Atherosclerosis to Treatment with Fluvastatin According to Genotypes of rs2383206 SNP at the 9p21 Locus

	**Genotypes**			
	**GG**	**GA**	**AA**	**P**

**A. Response of plasma lipids levels**				

N = 165	51	97	17	

% Change in total cholesterol	-14.7 ± 10.7	-16.5 ± 14.9	-15.4 ± 10.6	0.183*

% Change in LDL-C	-25.8 ± 13.8	-26.1 ± 20.2	-23.2 ± 14.5	0.040*

% Change in HDL-C	9.2 ± 14.6	9.9 ± 16.1	6.7 ± 15.0	0.734

% Change in Triglyceride	9.6 ± 42.9	5.4 ± 36.0	5.1 ± 24.8	0.002*

% Change in apoB	-16.4 ± 14.0	-16.9 ± 17.1	-13.6 ± 11.6	0.737

% Change in apo A1	8.1 ± 16.5	6.4 ± 22.0	4.5 ± 16.0	0.801

**B. Response of coronary atherosclerosis**				

**N **= 147	46	84	17	

New coronary lesions (mean)	0.30 ± 0.47	0.25 ± 0.45	0.24 ± 0.44	0.768

Subjects with new coronary lesions (%)	14 (30.4)	21 (25.0)	4 (23.5)	0.664

New coronary occlusions (mean)	0.04 ± 0.21	0.04 ± 0.19	0.0 ± 0.0	0.698

Subjects with new coronary occlusions (%)	2 (4.4)	3 (3.7)	0 (0)	0.694

Average final MLD (mm)	1.54 ± 0.44	1.60 ± 0.47	1.76 ± 0.50	0.396

Change in MLD (mm)	-0.04 ± 0.27	-0.05 ± 0.26	-0.01 ± 0.20	0.807

**Abbreviations: **m: meter; Kg: Kilogram; mmHg: millimeter mercury; mg/dl: milligram per deciliter; HDL-C: High-density lipoprotein-cholesterol; LDL-C: low-density lipoprotein-cholesterol; Apo A: apolipoprotein A; Apo B: apolipoprotein B; Apo C-III: apolipoprotein C3; MLD: minimal lumen diameter.

## Discussion

We determined the association of quantitative indices of severity of coronary atherosclerosis, as determined by serial QCA, with 4 SNPs at the 9p21 locus; previously shown to be associated with the risk of CAD in case-control GWAS [1-4,11,12]. We found no significant association between the quantitative indices of severity of coronary atherosclerosis and the SNPs at the 9p21 in the well-characterized LCAS population. The availability of serial QCA also provided the opportunity to examine the potential association of development of new coronary lesions and progression of the existing coronary lesions. We found no significant SNP and haplotype effect on the development of new coronary lesions and the progression of coronary atherosclerosis over a period of 2 1/2 years neither in the placebo group nor in the entire population when treatment with fluvastatin was included as a covariate. In contrast, plasma HDL-C levels and treatment with fluvastatin were the only independent predictors of progression of coronary atherosclerosis (inverse relationship). There were also no genotype-by-treatment interactions between the 4 SNPs at the 9p21 locus and the response of coronary atherosclerosis to treatment with fluvastatin. Moreover, we detected no significant association between the 9p21 locus SNPs and plasma levels of total cholesterol, HDL-C, LDL-C, triglycerides or apolipoproteins. The findings suggest absence of a strong association between the severity, development or progression of coronary atherosclerosis, as determined by QCA, and the 4 SNPs at the 9p21 locus.

The results of the present study is in accord with the results of a recent study showing no significant association between the severity of carotid atherosclerosis as determined by the measurement of carotid intima-media thickness by ultrasound and the 4 SNPs at the 9p21 locus [23]. The null results of these two studies should be weighed against the results of the case-control GWAS showing a strong association between the 9p21 locus and risk of CAD. The previous null study had a very large sample size [23] and low chance of type II statistical error. It is also noteworthy that the Ottawa Heart Study-1 and Ottawa Heart Study-2 each had a sample size of approximately 320 cases and controls [2]. Despite the modest sample sizes there were significant differences in the distribution of genotypes of the 9p21 locus SNPs between the cases and controls in both datasets [2]. Nonetheless, we considered the possibility of type II statistical error in the present study. Therefore, we calculated the power of the present study, post hoc, to detect differences in MLD, the primary quantitative phenotype of severity of coronary atherosclerosis. Accordingly, the number of observed genotypes in the study population provided 95.5%, 92.9% and 73.9% power to detect a 20% difference in the baseline MLD between subjects with the GG vs. GA, GA vs. AA and GG vs. AA genotypes, respectively, and 98.1% between GG vs. GA and AA genotypes, when α is set at 0.05. Likewise, the power to detect a 15% difference in mean MLD between GG vs. GA and AA genotypes was 86%. For a 10% or smaller differences in the mean MLD among the genotypes, the sample size of the present study did not offer sufficient power. Therefore, we can not exclude the possibility that the 9p21 locus imparts an effect on MLD that is less than 10% (0.165 mm). Hence, smaller differences among the genotypes are beyond the level of detection of the present study. The presence of undetected small differences in the MLD among the genotype may impart clinical significance and affect the clinical outcome. As for the progression of coronary atherosclerosis during the follow up period, the time interval between the two QCA was 2.5 years, which was sufficient to detect the effects of treatment and plasma HDL-C levels. Accordingly, one would have expected to detect the effects of SNPs at 9p21 locus on progression of coronary atherosclerosis if they were greater than the effects of treatment or plasma HDL-C levels. Smaller effects, however, could remain undetected. We also note that while QCA is a robust tool for the measurement of lumen diameter and area, but it may not be sufficiently sensitive to changes in plaque burden. As for the plasma LDL-C and HDL-C levels, the sample size of the study provided 80% power to detect approximately 8 mg/dl and 4 mg/dl differences between the two common genotypes of the SNPs, respectively. Overall, we noted no trend toward an association for any of the quantitative indices of severity of coronary atherosclerosis or plasma levels of lipid to suggest the possibility of type II statistical error. Finally, we note the prospective design of the present study wherein each individual was his/her own control as regards the progression of coronary atherosclerosis and the detailed phenotypic definition of atherosclerosis as a continuous phenotype as potential strengths of the present study.

Various phenotypes including ischemic stroke, abdominal aortic aneurysm, intra-cranial aneurysm, coronary artery calcification, osteoporosis, type 2 diabetes mellitus and possibly Alzheimer's disease have been associated with the 9p21 locus [5,24-28]. Many but not all of the associated phenotypes may share a common vascular etiology with atherosclerosis as an underpinning milieu. The 9p21 locus contains the tumor suppressor proteins p16INK4a and P16INK4b, which are frequently lost or mutated in various human cancers, such as in leukemia, glioma, head and neck cancers and bladder cancers [13-15]. The naturally occurring mutations at this locus are not known to predispose to premature atherosclerosis. Similarly, genetic deletion of the locus in the mouse is not known to predispose to or protect from atherosclerosis [16,17]. Accordingly, given that LD in the human genome can span large chromosomal segments [29], one may speculate that the 9p21 locus is in LD with a remote locus that serves as a true susceptibility locus for coronary atherosclerosis. Alternatively, the locus also contains a gene that transcribes a non-coding RNA, with yet to-be-defined function. Whether one of the two known genes or the non-coding RNA at the 9p21 locus or a distant locus/gene in LD with the 9p21 locus is responsible for the observed and consistent association of the 9p21 locus with CAD remains to be established.

The most significant impact of the results of the GWAS is likely to be in elucidation of the novel mechanistic pathways involved in the pathogenesis of atherosclerosis and its thrombotic complications. Such mechanistic studies could lead to identification of new therapeutic targets and the development of new drugs. In general, the clinical utility of the results of GWAS for risk stratification and prognostication is expected to be relatively modest; partly because of the low pre-test likelihood of the clinical events and relatively modest impact of the vast majority of the risk alleles for a complex trait. Therefore, delineation of molecular mechanisms by which the 9p21 locus imparts susceptibility to broadly defined CAD could have considerable clinical implications. An integral part of and likely a pre-requisite for the mechanistic studies pursuant to the results of the GWAS is identification of the precise phenotype of coronary heart disease that is associated with the 9p21 locus.

## Conclusion

The results of the present study show no significant association between the quantitative indices of severity of coronary atherosclerosis and the 4 SNPs at the 9p21 locus, previously identified as susceptibility SNPs for coronary heart disease in case-control GWAS. The null results of the present study along with the null results of a recent study showing no association between carotid intima-media thickness and the 9p21 locus [23] suggest that the observed association of 9p21 locus with CAD in the GWAS could be mediated through complex mechanisms beyond the severity or progression of atherosclerosis. We suggest mechanistic studies are needed to delineate the potential role of the SNPs at the 9p21 locus on plaque instability and intra-arterial thrombosis.

## Competing interests

The authors declare that they have no competing interests.

## Authors' contributions

SNC performed the genotyping. CMB participated in the design of the study and clinical phenotyping. AMG coordinated the clinical study and helped with clinical phenotyping. AJM conceived and designed the genetic studies, analyzed the data and wrote the manuscript. All authors read and approved the final manuscript.

## Pre-publication history

The pre-publication history for this paper can be accessed here:


